# Trade-Offs Between Hydraulic Efficiency and Safety in Cotton (*Gossypium hirsutum* L.) Stems Under Elevated CO_2_ and Salt Stress

**DOI:** 10.3390/plants14020298

**Published:** 2025-01-20

**Authors:** Qing Zhao, Jinliang Chen, Jian Kang, Shaozhong Kang

**Affiliations:** 1 State Key Laboratory of Efficient Utilization of Agricultural Water Resources, Beijing 100083, China; zhaoqing.chine@gmail.com (Q.Z.); chenjinliang@cau.edu.cn (J.C.); jkang@cau.edu.cn (J.K.); 2 National Field Scientific Observation and Research Station on Efficient Water Use of Oasis Agriculture in Wuwei of Gansu Province, Wuwei 733009, China; 3 Center for Agricultural Water Research in China, China Agricultural University, Beijing 100083, China

**Keywords:** elevated CO_2_, embolism, hydraulic conductance, cotton, salt stress, xylem anatomy

## Abstract

Plants respond to environmental changes by altering the anatomical structure of the xylem and its hydraulic properties. While numerous studies have explored the effects of individual environmental factors on crops, the combined interactions of these factors remain underexplored. As climate change intensifies, the occurrence of salt stress is becoming more frequent, alongside a rise in atmospheric CO_2_ concentration. This study aims to investigate the effects of elevated CO_2_ and salt stress on the hydraulic traits and xylem anatomical structures of cotton stems. Potted cotton plants were exposed to different CO_2_ concentrations (aC: 400 ppm; eC: 800 ppm) and salinity levels (aS: 0‰ soil salinity; eS: 6‰ soil salinity). The study found that under eC and eS conditions, a trade-off exists between hydraulic efficiency and safety in cotton stems, which may be partially attributed to xylem anatomical structures. Specifically, eS significantly reduced stem hydraulic conductivity under aC conditions and decreased vessel diameter but increased the proportion of small-diameter vessels and enhanced implosion resistance ((t/b)^2^), which strengthened the xylem’s resistance to salt-induced embolism. eC altered the response pattern of xylem hydraulic conductivity and embolism resistance to salt stress, with increased vessel diameter enhancing hydraulic conductivity but reducing xylem resistance to embolism. These findings enhance our comprehension of plant hydraulic adaptation under future climatic conditions and provide new insights into the trade-offs between xylem structure and function.

## 1. Introduction

The xylem plays a crucial role in terrestrial plants as the core structure for water transport. Its highly specialized vessel network ensures the continuous and efficient transport of water from roots to above-ground parts while minimizing energy loss, thereby supporting the water supply required for leaf gas exchange under various environmental conditions. The realization of this key function relies on the unique hydraulic properties of the xylem, which are essential for plant survival and productivity [1,2] and are strongly affected by vessel anatomy and environmental factors. Additionally, hydraulic traits vary significantly among species when adapting to diverse environments [3,4].

Among various environmental stresses, soil salinization stands out as a major challenge for plants and one of the most critical abiotic constraints on global agricultural productivity. Currently, salinization affects more than 6% of the world’s total land area and approximately 20% of irrigated land [5]. In saline soils, plants face osmotic stress and ion toxicity throughout their lifecycle, hindering the efficient establishment of their hydraulic transport system and inhibiting growth and development [6]. To cope with salt stress, plants exhibit adaptive changes in xylem structure, such as reduced vessel size, increased vessel number, and thickened vessel walls. These anatomical adjustments significantly impact xylem hydraulic conductivity and regulate water transport efficiency [6,7]. Studies have shown that these changes are closely associated with the intensity and duration of stress [8]. For instance, under salt stress, stem xylem vessel diameter significantly decreases, while vessel density increases, further enhancing plant adaptability to stressful environments. However, the greater the stress intensity, the more pronounced the impact on the xylem structure [6].

Meanwhile, being the primary component essential for photosynthesis, elevated atmospheric CO_2_ profoundly impacts plant physiological processes and growth dynamics. Atmospheric CO_2_ is projected to reach approximately 560 µmol mol⁻^1^ by 2050 and around 700 µmol mol⁻^1^ by 2100 [9]. Studies suggest that under elevated atmospheric CO_2_, plants may better cope with drought or salt-induced stress by reducing transpiration and increasing water potential [10,11,12]. However, it remains a topic of debate regarding the effects of elevated CO_2_ on the xylem of plant stems. One perspective posits that elevated CO_2_ notably increases xylem vessel diameter, hydraulic conductivity, and vulnerability to embolism [13,14,15]. Conversely, other studies have found that elevated CO_2_ has minimal or negligible effects on these traits [16,17,18]. Current research on stem xylem largely focuses on woody plants, with less attention given to crops. However, xylem hydraulic traits also play a crucial role in crop growth regulation [19,20]. Due to the relatively low construction cost of xylem, crops may be more sensitive to embolism caused by stress compared to woody plants [21,22]. However, research on the effects of elevated CO_2_ on crop stem xylem remains limited and controversial [23,24]. Therefore, it is essential to further explore the reaction mechanisms of crop xylem under elevated CO_2_ conditions, particularly its interactions with soil salinity stress.

Prior research has demonstrated that xylem anatomy is closely related to plant functions, as it mediates the trade-off between xylem water transport efficiency and safety by regulating the water conduction of different tissues and the leaf gas exchange processes [25]. Additionally, Baas et al. [26] proposed the “trade-off triangle” theory involving xylem transport efficiency, safety, and mechanical strength. For instance, the presence of narrower conduits can serve to mitigate the risk of embolism, despite this often coming at the cost of reduced water transport efficiency [14,27]. Many studies have found that plants under salinity stress typically exhibit smaller conduits, which enhance xylem embolism resistance, reflecting an adaptive strategy to keep the water supply needed for plant growth in stressful environments [28,29]. However, studies on the trade-off between xylem hydraulic efficiency and safety remain inconclusive. Maherali et al. [30] reported in a study of 167 species that the trade-off between hydraulic efficiency and embolism resistance exhibited only a slight correlation. Zhang et al. [31] found that the trade-off between hydraulic efficiency and safety was weak or even absent in large lianas and woody plants. Furthermore, this relationship may disappear due to xylem over-construction, as in Podocarpaceae species, which have high xylem density and implosion resistance but show a decoupling between embolism resistance and hydraulic efficiency [32].

Current research on plant hydraulic traits and embolism vulnerability mainly focuses on their seasonal variations [33,34,35] or performance in large-scale community patterns [36]. However, there is limited research on the effects of elevated CO_2_ combined with salt stress on hydraulic traits and vulnerability to embolism of crops in agricultural ecosystems [2]. Cotton, as one of the most important fiber crops in the world, plays a crucial role in agricultural production and serves as an economic backbone for many countries. Cotton is widely cultivated around the world, particularly in the arid and semi-arid regions of Asia, Africa, and the Americas, where cotton is one of the main economic crops [37]. In China, cotton production is concentrated in the arid and semi-arid regions of the northwest, where salinization poses a significant threat to cotton growth. Due to factors such as over-irrigation, poor drainage, and climate change, soil salinity levels are rising in these cotton-growing areas, severely threatening cotton growth and yield [38]. To this end, this study focuses on the C3 crop cotton (*Gossypium hirsutum* L.), aiming to (1) determine the impacts of elevated CO_2_ and salinity stress on stem hydraulic efficiency and safety in cotton, and (2) investigate the trade-off between hydraulic efficiency and safety in cotton, analyzing how this trade-off is mediated through xylem anatomical traits.

## 2. Results

### 2.1. Stem Hydraulic Efficiency

**Figure 1:**
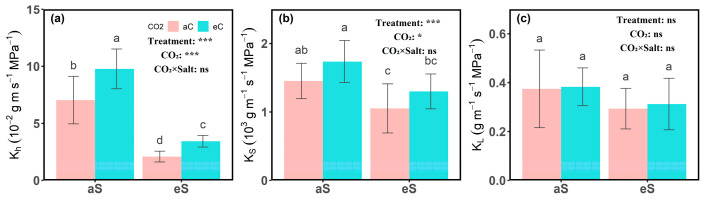
(**a**) Stem hydraulic conductivity (K_h_), (**b**) specific hydraulic conductivity (K_S_), and (**c**) leaf-specific hydraulic conductivity (K_L_) under different CO_2_ and salinity treatments in cotton (*Gossypium hirsutum* L.). Different letters indicate significant differences between treatments (*p* < 0.05). Error bars represent the mean ± SE. Significance levels are indicated as follows: *, *p* < 0.05; ***, *p* < 0.001; ns, not significant.

Under aC conditions, eS treatment significantly reduced stem hydraulic conductivity (K_h_) and specific hydraulic conductivity (K_S_) (*p*  <  0.05). Specifically, K_h_ and K_S_ decreased from 0.070 ± 0.021 g m s^−1^ MPa^−1^ and 1450 ± 259 g m^−1^ s^−1^ MPa^−1^ under aS treatment to 0.021 ± 0.004 g m s^−1^ MPa^−1^ and 1050 ± 358 g m^−1^ s^−1^ MPa^−1^ under eS treatment (Figure 1a,b). eC conditions significantly increased K_h_ and K_S_, with K_h_ increasing by 38.92% and 64.11% under aS and eS treatments, respectively, and K_S_ increasing by 20% and 23.81% compared to aC conditions. In terms of leaf-specific hydraulic conductivity (K_L_), this study found no significant impact of elevated CO_2_ and salinity stress on K_L_ (Figure 1c).

### 2.2. Vulnerability Analysis

**Figure 2:**
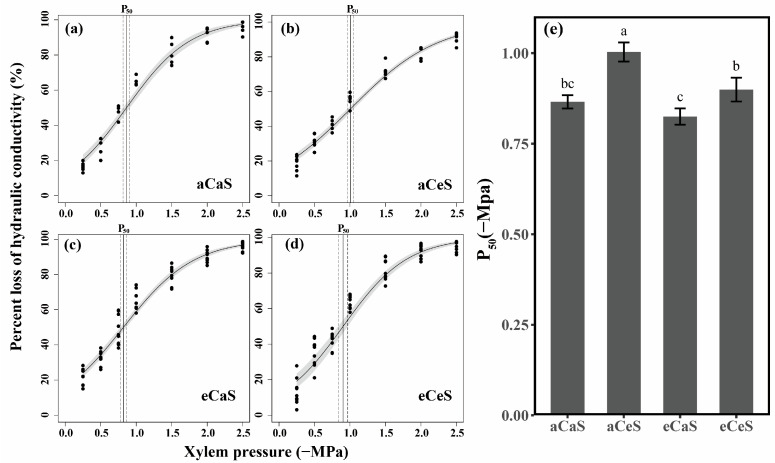
Stem vulnerability curves under aCaS (**a**), aCeS (**b**), eCaS (**c**), and eCeS (**d**) treatments in cotton (*Gossypium hirsutum* L.). (**e**) Water potential at 50% loss of hydraulic conductivity (P50) under different treatments. In panels (**a**–**d**), the black dots represents the percent of hydraulic conductivity loss at the corresponding water potential, the gray shadow represents the 95% confidence interval of the fitted line, and the vertical dashed line indicates P_50_. Different letters in the histograms indicate significant differences in P_50_ between treatments (*p* < 0.05).

eS treatment significantly decreased the water potential at 50% loss of stem hydraulic conductivity (P_50_). Under aC conditions, P_50_ decreased from −0.75 ± 0.11 MPa under aS treatment to −0.86 ± 0.06 MPa under eS treatment, indicating that salinity stress reduced the vulnerability of the stem xylem to embolism (Figure 2). Under eC conditions, P_50_ increased for both aS and eS treatments, indicating that elevated CO_2_ increased the vulnerability of the stem xylem to embolism.

### 2.3. Anatomical Characteristics

**Figure 3:**
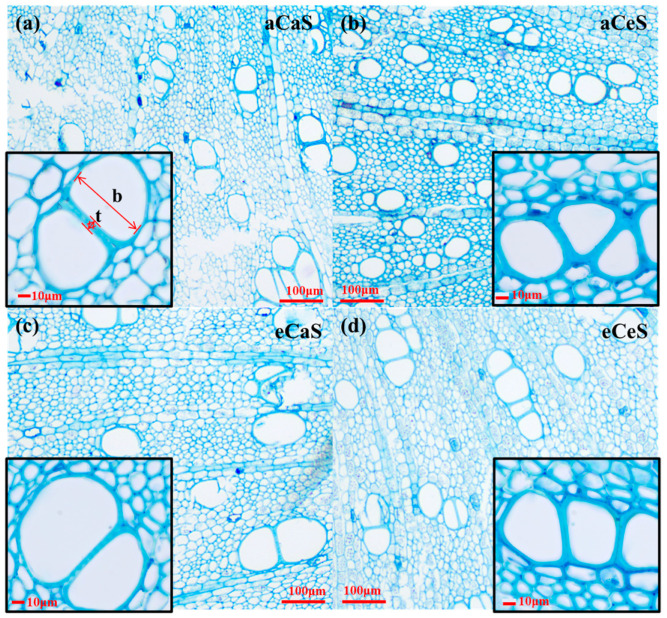
(**a**–**d**) Transverse microscopic stem sections of the cotton (*Gossypium hirsutum* L.) under different CO_2_ and salinity treatments. t is the thickness of two adjacent vessel walls, and b is the vessel diameter. Scale bars = 100 µm, and 10 µm for insets.

**Figure 4:**
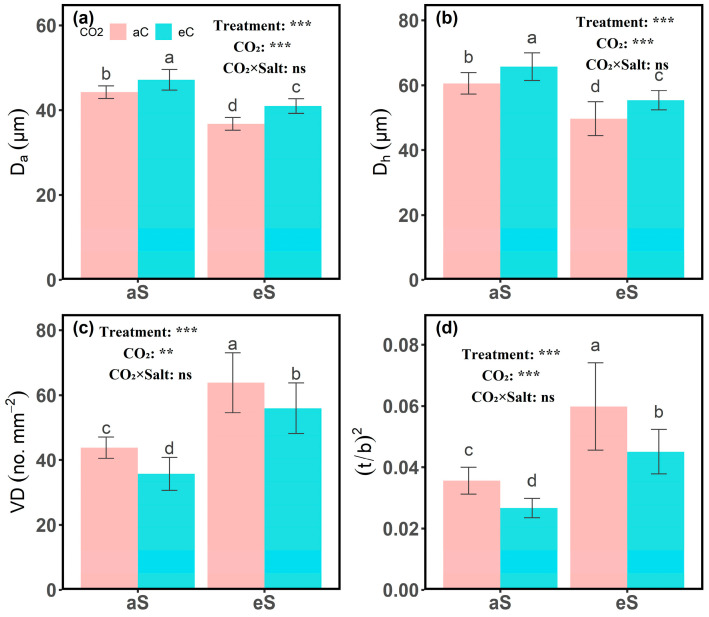
(**a**) Anatomical diameter (D_a_), (**b**) hydraulic diameter (D_h_), (**c**) vessel density (VD), and (**d**) implosion resistance ((t/b)^2^) under different CO_2_ and salinity treatments in cotton (*Gossypium hirsutum* L.). Different letters indicate significant differences between treatments (*p* < 0.05). Error bars represent the mean ± SE. Significance levels are indicated as follows: **, *p* < 0.01; ***, *p* < 0.001; ns, not significant.

**Figure 5:**
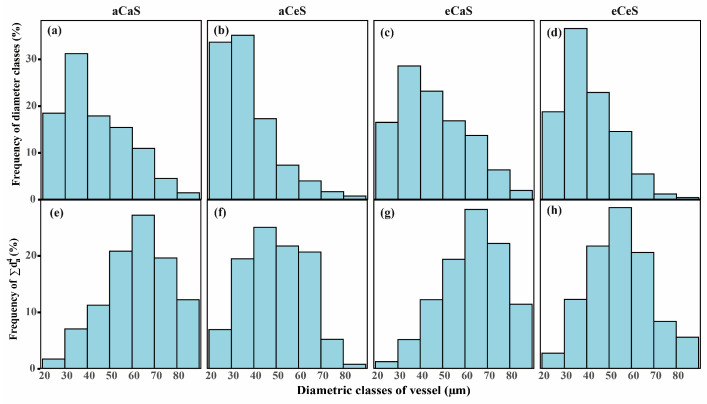
Percentage of vessels with diameters of 10 µm under CO_2_ and salinity treatments in cotton (*Gossypium hirsutum* L.). (**a**–**d**) Percentage based on total vessel number under different treatments; (**e**–**h**) percentage based on the sum of the fourth power of all vessel diameters under different treatments. This reflects the relative hydraulic importance of each diameter class based on the Hagen–Poiseuille law.

The effects of CO_2_ and salinity treatments on stem anatomical traits and vessel distribution are shown in Figure 3, Figure 4 and Figure 5. Under aC conditions, eS treatment significantly reduced the anatomical diameter (D_a_) and hydraulic diameter (D_h_) of stem vessels. Specifically, D_a_ decreased from 44.2 ± 1.5 μm under aS treatment to 36.8 ± 1.5 μm under eS treatment, and D_h_ decreased from 60.6 ± 3.3 μm under aS treatment to 49.7 ± 5.2 μm under eS treatment (Figure 4a,b). In contrast, vessel density (VD) significantly increased under eS treatment (Figure 4c). The vessel diameter distribution charts (Figure 5a,b) show that compared with aS treatment, eS treatment decreased the proportion of larger vessels and increased the proportion of smaller vessels in the stem. Furthermore, the predicted hydraulic conductivity charts for each diameter class (Figure 5e,f) show that the proportion of hydraulic conductivity from smaller vessel sizes increased under eS treatment.

Under eC conditions, D_a_ and D_h_ significantly increased. Compared to aC conditions, D_a_ increased by 6.78% and 11.41% under aS and eS treatments, respectively, while D_h_ increased by 8.42% and 11.47% under aS and eS treatments, respectively. Meanwhile, VD significantly decreased under eC conditions. From the vessel diameter distribution charts (Figure 5c,d), it can be seen that under eC conditions, the proportion of larger vessels in the stem increased, while the proportion of smaller vessels decreased. The predicted hydraulic conductivity charts for each diameter class (Figure 5g,h) show that the proportion of hydraulic conductivity from larger vessel sizes increased under eC treatment. In addition, (t/b)^2^ represents the implosion resistance of the plant xylem cell wall. Under eS treatment, (t/b)^2^ significantly increased, while under eC conditions, (t/b)^2^ showed a decreasing trend (Figure 4d).

### 2.4. Tradeoffs Between Xylem Hydraulic Efficiency and Safety

**Figure 6:**
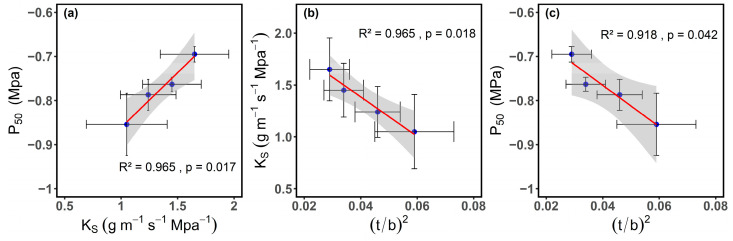
(**a**) Stem-specific hydraulic conductivity (K_S_) and water potential at 50% conductivity loss (P_50_) in cotton (*Gossypium hirsutum* L.), (**b**) implosion resistance ((t/b)^2^) and stem-specific hydraulic conductivity (K_S_), and (**c**) the relationship between (t/b)^2^ and P_50_. Error bars represent means ± SE. The red solid line represents linear fitting, and the gray area represents the confidence interval.

P_50_ increases with the increase in K_S_ (Figure 6a), indicating a trade-off between hydraulic efficiency and hydraulic safety in the xylem. As K_S_ decreases, the stem’s resistance to embolism increases. As (t/b)^2^ increases, K_S_ decreases, suggesting that the enhanced mechanical strength of the vascular wall limits the water conduction capacity of the xylem (Figure 6b). Additionally, P_50_ decreases with the increase in (t/b)^2^, and exhibits a negative correlation, indicating that anatomical traits can influence the occurrence of cavitation to a certain degree, consequently impacting the stem’s embolism resistance and achieving a trade-off between hydraulic efficiency and safety (Figure 6c).

## 3. Discussion

### 3.1. Xylem Hydraulic Efficiency Under Elevated CO_2_ and Salt Stress

Salt stress severely limits the number and size of xylem vessels, thereby restricting the development of the plant’s efficient transport system [39,40]. In this study, salt stress caused a decrease in K_h_ and K_S_, as well as a reduction in D_a_ and D_h_ (Figure 1 and Figure 2), indicating that the plant’s hydraulic function was severely limited. However, eC alleviated this limitation to some extent, as evidenced by the increase in K_h_, K_s_, D_a_, and D_h_. Recent studies have also confirmed that plants exposed to eC show an overall increase in vessel diameter and xylem hydraulic conductivity [41,42]. This change may be related to the effect of elevated CO_2_ stimulating stem volume growth, which leads to an overall increase in vessel diameter [43]. The above results suggest that plants can adapt to different environmental conditions by adjusting their hydraulic structure.

The small Da under eS may limit water transport capacity, as reflected in the suppressed K_h_ and K_S_. Xylem hydraulic conductivity largely depends on vessel diameter and number [44], and the decrease in hydraulic efficiency may be directly related to the limitations of Dh and Da (Figure 4). Under eS, the vessel diameter decreases while VD increases, which is consistent with previous findings [45] and corroborates the theory that plants exposed to stress conditions face a higher embolism risk. They may mitigate this risk by reducing vessel diameter, even though such a change may result in decreased hydraulic efficiency [36]. Consequently, decreasing the proportion of wide vessels while increasing VD might be viewed as a strategic adjustment made by plants to adapt to salt stress. While this adjustment may reduce the hydraulic capacity of individual vessels due to smaller diameters, the increase in vessel density helps to compensate for this reduction, allowing the plant to maintain overall water transport capacity while reducing the risk of embolism. Compared to the aCeS treatment, wider vessels under eCeS seem to be the optimal strategy for regulating xylem hydraulic conductivity. This change helps maintain marginal transpiration flux in stressful environments and may prevent hydraulic failure by improving hydraulic conductivity and reducing tension within the xylem [46].

### 3.2. Xylem Hydraulic Safety Under Elevated CO_2_ and Salt Stress

Generally, high salt concentrations reduce cell water potential, further increasing negative pressure in the xylem, which accelerates cavitation formation and embolism occurrence. In addition, salt stress may cause plants to lose more water during water transport, further exacerbating the risk of embolism. The P_50_ value is a crucial indicator for evaluating the plant’s resistance to embolism [47,48]. In general, plants with lower P50 values have stronger resistance to external stresses and longer survival times [49,50]. This study found that under eS, P_50_ significantly decreased, indicating enhanced resistance to embolism, which is consistent with previous studies on trees or crops under salt stress, where stem embolism resistance was enhanced under salt stress [14,27]. In addition, the P_50_ values in this study are also close to those reported in other research on cotton (*Gossypium hirsutum* L.) P_50_ values [51].

This study also found that under eC, the embolism vulnerability of the stem increased, as P50 occurred at a higher water potential. This is also consistent with some previous studies suggesting that plants growing under eC may have lower resistance to embolism [14,52,53], though this has not been widely substantiated through experimental findings. In fact, the reaction in the xylem embolism threshold to eC is a considerable variable [54,55,56,57]. For example, Domec et al. [54] found that two diffuse-porous angiosperms growing under eC exhibited a smaller negative P_50_, whereas this trait in one non-porous gymnosperm and one ring-porous angiosperm was unaffected by CO_2_. According to a recent investigation conducted by Hao et al. [55], the P_50_ response to elevated CO_2_ showed almost no change across six species. On the other hand, compared to aCaS, when CO_2_ is coupled with salt stress, CO_2_ partially mitigates the synergistic effect of salt stress on embolism resistance, as indicated by the increase in P_50_ values.

Changes in the vulnerability of the xylem to embolism may be closely related to changes in its structural characteristics. Generally, smaller vessels are better at resisting cavitation than larger ones because the pit membrane surface area on small vessels is smaller, and the likelihood of large pores forming is lower, thus reducing the risk of bubbles passing through pits and triggering cavitation and embolism [58]. In addition, smaller diameters increase the structural strength of the cell wall to prevent collapse [59]. Therefore, a higher proportion of small vessels under eS conditions can reduce the likelihood of embolism in the xylem to some extent, whereas an increase in vessel size under eC conditions may increase embolism risk. Additionally, (t/b)^2^ reflects the ability in the cell wall’s resistance to resist flexural stress resulting from pressure variations between neighboring vessels [60]. The increase in (t/b)^2^ under salt stress further indicates enhanced cavitation resistance, thereby improving the hydraulic safety of the xylem.

### 3.3. Trade-Off Between Hydraulic Efficiency and Embolism Resistance and How Anatomy Traits Influence This Trade-Off

It is generally believed that there is a clear trade-off between the efficiency and safety of xylem water transport, either within or between species. A decrease in the average diameter of xylem vessels reduces the hydraulic conductivity of the xylem, while simultaneously enhancing embolism resistance, because smaller vessels are less affected by cavitation [61]. However, some previous studies have not explicitly supported this trade-off relationship [62,63,64]. This study found that K_S_ is significantly correlated with P_50_, further proving that there is indeed a trade-off between hydraulic efficiency and safety in the xylem of cotton.

To adapt to salt stress, plants typically adjust their vessel structure, producing more but narrower vessels. Although this adjustment reduces water transport efficiency, the increase in vessel density can enhance hydraulic safety [65,66]. The increase in vessel number means that even if some vessels become embolized, the remaining functional vessels can provide backup water flow paths, thereby enhancing xylem redundancy [67,68]. This feature helps reduce the risk of functional loss and plant mortality at a certain embolism rate [65,66,69]. When elevated CO_2_ combines with salt stress, while hydraulic conductivity is somewhat mitigated, embolism resistance also declines. This reflects cotton’s strategy of optimizing resource allocation and functional coordination to achieve a balance between hydraulic efficiency and safety in response to environmental changes.

Our results also show that stem hydraulic conductivity is significantly negatively correlated with (t/b)^2^ and significantly positively correlated with P50, which is consistent with previous research [70]. Under low water potential (high tension), vessels require stronger structural reinforcement to avoid implosion [71]. When embolism occurs, the vessel walls experience greater bending stress due to the pressure gradient between adjacent vessels, and a higher (t/b)^2^ can protect vessels from damage under stress [71,72]. These findings suggest that, while water transport efficiency decreases, the mechanical strength of the stem is enhanced, thus reducing the risk of embolism formation [73]. Therefore, (t/b)^2^ plays an important role in cotton’s hydraulic regulation under changing environments, and by adjusting (t/b)^2^, plants can achieve a balance between hydraulic efficiency and safety. Therefore, combining the trends of vessel diameter, vessel density, vessel distribution, and (t/b)^2^ changes in this study, we conclude that cotton effectively responds to embolism vulnerability caused by environmental changes through adjustments in xylem anatomical features. This adaptive strategy provides a structural foundation and functional support for cotton’s survival under complex stress conditions.

## 4. Materials and Methods

### 4.1. Plant Materials and Growth Conditions

The experiment was conducted from April to August 2021 in a natural supplementary light artificial climate chamber at the National Field Scientific Observation and Research Station on Efficient Water Use of Oasis Agriculture in Wuwei of Gansu Province, China (N 37°52′, E 102°50′, altitude 1581 m). Cotton (*Gossypium hirsutum* L. Cv, Xinluzao72) seeds were sown in seedling trays containing nutrient soil and cultivated in the trays for six weeks. The seeds were moistened with distilled water and placed in an artificial climate chamber with supplementary natural lighting. The chamber conditions were set to a temperature of 25 °C during the day and 18 °C at night, with a relative humidity of 60–70%. In the seventh week, the seedlings were transplanted into 11 L plastic pots filled with local sandy loam, with the surface layer mixed with nutrient soil to decrease irrigation impact and prevent soil compaction. Each pot was planted with one cotton seedling, and the potted plants were evenly distributed across four climate chambers. The experiment followed a 2 × 2 factorial design to explore the main effects and interactions of CO_2_ and salinity on the hydraulic and anatomical traits of cotton plants. Among the four climate chambers, two were maintained at ambient CO_2_ concentration (aC: 400 ppm), while the other two were set at elevated CO_2_ concentration (eC: 800 ppm). Under each CO_2_ treatment, a no-salinity treatment (aS, soil salinity 0‰) and a salinity treatment (eS, soil salinity 6‰) were applied. The salinity treatments were prepared by mixing salt (NaCl, MgSO_4_, and CaSO_4_ in a 2:2:1 mass ratio) with local soil, based on the chemical composition of local groundwater. All treatments maintained soil moisture at 70–90% of field capacity throughout the experiment. The growth chamber temperatures were maintained at 28 °C/20 °C (daytime [8:00 a.m.–6:00 p.m.]/nighttime [6:00 p.m.–8:00 a.m.]) with humidity levels of 60%/80%. Refer to [74] for more details on the equipment and control systems of the artificial climate chambers.

### 4.2. Stem Hydraulic Conductivity

During the cotton boll period (70 days post-transplanting), main stems from the middle part of the plant were collected before dawn, rapidly trimmed underwater, and then wrapped in black plastic bags and brought back to the laboratory. Each treatment included 12 replicates (2 CO_2_ chambers × 6 plants). Before measurement, both ends of the stems were trimmed underwater, and segments about 20 cm long were selected. The stems were initially flushed with a solution (20 mM KCl + 1 mM CaCl_2_) under 10 kPa pressure to remove in situ embolisms. Then, one end of the stem segment was attached to a 50–60 kPa gravity water head, and the other end was used for collecting the effluent. The outflowing water was measured using an analytical balance, collecting effluent every 1 min. Once the flow rate stabilized, the water flux (Q, g/min) was recorded to calculate the maximum hydraulic conductivity, which is defined as stem hydraulic conductance (K_h_, g m s^−1^ MPa^−1^).

(1) $$K_{h}=\frac{Q\times l}{\Delta P}$$

where, ΔP represents the static water pressure head applied, and l is the length of the stem segment.

Specific hydraulic conductivity (K_S_, g m^−1^ s^−1^ MPa^−1^) is calculated as the ratio of K_h_ to the stem cross-sectional area (SA, m^−2^):

(2) $$K_{S}=\frac{K_{h}}{SA}$$

Leaf-specific hydraulic conductivity (K_L_, g m^−1^ s^−1^ MPa^−1^) is calculated as the ratio of K_h_ to leaf area (LA, m^−2^):

(3) $$K_{L}=\frac{K_{h}}{LA}$$

Leaf area is the total leaf area supported above the stem segment. Both leaf area and stem cross-sectional area were measured using ImageJ software (version 1.54k), (National Institutes of Health, Bethesda, MD, USA).

### 4.3. Stem Vulnerability Curve

The stem vulnerability curve (VC) was measured using the air injection method [73]. After measuring K_h_, the stem segment was placed into a pressure chamber and pressurized, where it was pressurized to 0.25, 0.5, 0.75, 1, 1.5, 2, and 2.5 MPa for 10 min at each pressure point to induce embolism formation. The pressure was gradually increased to these values in steps, creating a pressure gradient. After stopping the pressure, the stem was equilibrated for 15 min before measuring the corresponding hydraulic conductivity (K_hi_) at each pressure level. This process continued until the hydraulic conductivity loss (PLC, %) reached over 80%. The relationship curve between the applied pressure (P) and the corresponding PLC represents the stem vulnerability curve. The PLC is calculated as follows:

(4) $$PLC=1-\frac{K_{hi}}{K_{h}}\times 100$$

The vulnerability curve is fitted using an exponential S-shaped function [75]:

(5) $$PLC=\frac{100}{1+e^{a(\psi -b)}}$$

where a represents the slope of the curve, and b represents the water potential corresponding to a 50% loss in hydraulic conductivity.

### 4.4. Stem Xylem Anatomy

Cross-sections of the stems used to measure hydraulic conductivity were taken, preserved in FAA fixative, dehydrated in a graded series of tert-butyl alcohol, and infiltrated with paraffin at 56–58 °C. Sections of 10 µm thickness were obtained using a rotary microtome (Leitz, Wetzlar, Germany). The slides were dewaxed, rehydrated, and stained with Toluidine Blue in 95% ethanol. The sections were then photographed using a digital optical microscope (BA210; Motic, Xiamen, China). The number and diameter of vessels in each stem were measured. The diameter (anatomical diameter; D_a_) was calculated as the equivalent circular diameter. Variables characterizing the stem xylem anatomy include vessel density (VD, vessel number per mm^2^), average vessel diameter, predicted hydraulic conductivity, and estimated average hydraulic diameter. According to the Hagen–Poiseuille law [76,77], the sum of the fourth power of all vessel diameters (∑D_a_⁴) was used to predict hydraulic conductivity. The frequency distribution of Da classes for each treatment was determined. At the same time, the contribution percentage of each diameter class to overall water conductivity was calculated. Hydraulic diameter (D_h_) was calculated by weighting the contribution of each vessel to ∑D_a_⁴ using the relationship ∑D_a_⁵/∑D_a_⁴ [76,78]. The implosion resistance ((t/b)^2^) is a parameter that characterizes vessel structural strength [71], where t is the thickness of two adjacent vessel walls, and b is the vessel diameter (see Figure 3a for insets). Implosion resistance was measured for each diameter class, and the average value was calculated across all diameter classes.

### 4.5. Statistical Analysis

All data and statistical analyses in this study were performed in the R 4.3.2 statistical computing environment [79]. The stem VC curve was fitted using a sigmoidal model with the fitplc package in R [80]. The water potential (P_50_, -MPa) causing a 50% loss in xylem hydraulic conductivity was extracted from the fitted VC curve. The effects of CO_2_ and salinity on plant hydraulic and anatomical traits were evaluated using a two-way analysis of variance (ANOVA), and the impact of salinity under each CO₂ treatment was analyzed using a t-test. Differences were considered statistically significant when *p* ≤ 0.05.

## 5. Conclusions

Assessing how plants respond to multiple environmental change factors is essential for predicting future crop performance. In this study, we analyzed changes in stem hydraulic efficiency and embolism resistance of cotton (*Gossypium hirsutum* L.) under elevated CO_2_ concentrations and salt stress and explored how these hydraulic traits are regulated through anatomical structures. The results indicate that hydraulic efficiency significantly decreases under salt stress, while embolism resistance increases; in contrast, elevated CO_2_ concentrations improve hydraulic efficiency but decrease embolism resistance. This result further supports the trade-off between hydraulic efficiency and hydraulic safety, and this change may be related to anatomical adjustments in the xylem. Changes in hydraulic conductivity are closely related to changes in vessel characteristics. Under salt stress, smaller vessel diameters resulted in reduced water transport efficiency, but the increased proportion of smaller vessels and enhanced implosion resistance reflect increased embolism resistance. In contrast, elevated CO_2_ concentrations improved hydraulic efficiency but simultaneously weakened embolism resistance in the xylem, indicating that higher hydraulic efficiency may come at the cost of hydraulic safety. The findings of this research deepen our understanding of cotton’s (*Gossypium hirsutum* L.) hydraulic adaptation mechanisms in response to future climatic conditions and offer new perspectives on the trade-offs between xylem structure and function. This research not only clarifies the hydraulic strategies of cotton (*Gossypium hirsutum* L.) under complex environmental conditions but also provides a theoretical basis and practical insights for improving crop adaptability.

## Data Availability

Dataset available on request from the authors.

## References

[1] Venturas M.D., Sperry J.S., Hacke U.G. (2017). Plant xylem hydraulics: What we understand, current research, and future challenges. J. Integr. Plant Biol..

[2] Torres-Ruiz J.M., Cochard H., Delzon S., Boivin T., Burlett R., Cailleret M., Corso D., Delmas C.E.L., De Caceres M., Diaz-Espejo A. (2024). Plant hydraulics at the heart of plant, crops and ecosystem functions in the face of climate change. New Phytol..

[3] Butto V., Rozenberg P., Deslauriers A., Rossi S., Morin H. (2021). Environmental and developmental factors driving xylem anatomy and micro-density in black spruce. New Phytol..

[4] Swidrak I., Gruber A., Kofler W., Oberhuber W. (2011). Effects of environmental conditions on onset of xylem growth in *Pinus sylvestris* under drought. Tree Physiol..

[5] Munns R., Tester M. (2008). Mechanisms of salinity tolerance. Annu. Rev. Plant Biol..

[6] Jerszurki D., Sperling O., Parthasarathi T., Lichston J.E., Yaaran A., Moshelion M., Rachmilevitch S., Lazarovitch N. (2020). Wide vessels sustain marginal transpiration flux and do not optimize inefficient gas exchange activity under impaired hydraulic control and salinity. Physiol. Plant..

[7] Barzegargolchini B., Movafeghi A., Dehestani A., Mehrabanjoubani P. (2017). Morphological and anatomical changes in stems of Aeluropus littoralis under salt stress. J. Plant Mol. Breed..

[8] Nguyen H.T., Stanton D.E., Schmitz N., Farquhar G.D., Ball M.C. (2015). Growth responses of the mangrove Avicennia marina to salinity: Development and function of shoot hydraulic systems require saline conditions. Ann. Bot..

[9] NASA Global Climate Change: Vital Signs of the Planet. https://climate.nasa.gov/vital-signs/carbon-dioxide/?intent=111.

[10] Rozema J. (1993). Plant Responses to Atmospheric Carbon Dioxide Enrichment: Interactions with Some Soil and Atmospheric Conditions. Vegetatio.

[11] Robredo A., Pérez-López U., de la Maza H.S., González-Moro B., Lacuesta M., Mena-Petite A., Muñoz-Rueda A. (2007). Elevated CO_2_ alleviates the impact of drought on barley improving water status by lowering stomatal conductance and delaying its effects on photosynthesis. Environ. Exp. Bot..

[12] Pérez-López U., Robredo A., Lacuesta M., Mena-Petite A., Muñoz-Rueda A. (2009). The impact of salt stress on the water status of barley plants is partially mitigated by elevated CO_2_. Environ. Exp. Bot..

[13] Liu J., Kang S., Davies W.J., Ding R. (2019). Elevated [CO_2_] alleviates the impacts of water deficit on xylem anatomy and hydraulic properties of maize stems. Plant Cell Environ..

[14] Liu J., Hochberg U., Ding R., Xiong D., Dai Z., Zhao Q., Chen J., Ji S., Kang S. (2024). Elevated CO_2_ concentration increases maize growth under water deficit or soil salinity but with a higher risk of hydraulic failure. J. Exp. Bot..

[15] Kim K., Labbe N., Warren J.M., Elder T., Rials T.G. (2015). Chemical and anatomical changes in Liquidambar styraciflua L. xylem after long term exposure to elevated CO_2_. Environ. Pollut..

[16] Vaz M., Cochard H., Gazarini L., Graça J., Chaves M.M., Pereira J.S. (2012). Cork oak (*Quercus suber* L.) seedlings acclimate to elevated CO_2_ and water stress: Photosynthesis, growth, wood anatomy and hydraulic conductivity. Trees.

[17] Kilpeläinen A., Gerendiain A.Z., Luostarinen K., Peltola H., Kellomäki S. (2007). Elevated temperature and CO_2_ concentration effects on xylem anatomy of Scots pine. Tree Physiol..

[18] Kostiainen K., Kaakinen S., Saranpää P., Sigurdsson B.D., Linder S., Vapaavuori E. (2004). Effect of elevated [CO_2_] on stem wood properties of mature Norway spruce grown at different soil nutrient availability. Global Change Biol..

[19] Sperry J.S., Stiller V., Hacke U.G. (2003). Xylem hydraulics and the soil–plant–atmosphere continuum: Opportunities and unresolved issues. Agron. J..

[20] Stiller V., Lafitte H.R., Sperry J.S. (2003). Hydraulic properties of rice and the response of gas exchange to water stress. Plant Physiol..

[21] Holloway-Phillips M.M., Brodribb T.J. (2011). Minimum hydraulic safety leads to maximum water-use efficiency in a forage grass. Plant Cell Environ..

[22] Neufeld H.S., Grantz D.A., Meinzer F.C., Goldstein G., Crisosto G.M., Crisosto C. (1992). Genotypic variability in vulnerability of leaf xylem to cavitation in water-stressed and well-irrigated sugarcane. Plant Physiol..

[23] Medeiros J.S., Ward J.K. (2013). Increasing atmospheric [CO_2_] from glacial to future concentrations affects drought tolerance via impacts on leaves, xylem and their integrated function. New Phytol..

[24] Rico C., Pittermann J., Polley H.W., Aspinwall M.J., Fay P.A. (2013). The effect of subambient to elevated atmospheric CO_2_ concentration on vascular function in Helianthus annuus: Implications for plant response to climate change. New Phytol..

[25] Choat B., Medek D.E., Stuart S.A., Pasquet-Kok J., Egerton J.J., Salari H., Sack L., Ball M.C. (2011). Xylem traits mediate a trade-off between resistance to freeze-thaw-induced embolism and photosynthetic capacity in overwintering evergreens. New Phytol..

[26] Baas P., Ewers F.W., Davis S.D., Wheeler E.A., Hemsley A.R., Poole I. (2004). Evolution of xylem physiology. The Evolution of Plant Physiology.

[27] Beckett H.A.A., Bryant C., Neeman T., Mencuccini M., Ball M.C. (2024). Plasticity in branch water relations and stem hydraulic vulnerability enhances hydraulic safety in mangroves growing along a salinity gradient. Plant Cell Environ..

[28] Dai Y., Wang L., Wan X. (2020). Frost fatigue and its spring recovery of xylem conduits in ring-porous, diffuse-porous, and coniferous species in situ. Plant Physiol. Biochem..

[29] Fisher J.B., Goldstein G., Jones T.J., Cordell S. (2007). Wood vessel diameter is related to elevation and genotype in the Hawaiian tree Metrosideros polymorpha (Myrtaceae). Am. J. Bot..

[30] Maherali H., Pockman W.T., Jackson R.B. (2004). Adaptive variation in the vulnerability of woody plants to xylem cavitation. Ecology.

[31] Zhang L., Chen Y., Ma K., Bongers F., Sterck F.J. (2019). Fully exposed canopy tree and liana branches in a tropical forest differ in mechanical traits but are similar in hydraulic traits. Tree Physiol..

[32] Brodribb T.J. (2011). A functional analysis of podocarp ecology. Ecol. Podocarpaceae Trop. For..

[33] Losso A., Sailer J., Bar A., Ganthaler A., Mayr S. (2020). Insights into trunks of Pinus cembra L.: Analyses of hydraulics via electrical resistivity tomography. Trees.

[34] Martorell S., Medrano H., Tomas M., Escalona J.M., Flexas J., Diaz-Espejo A. (2015). Plasticity of vulnerability to leaf hydraulic dysfunction during acclimation to drought in grapevines: An osmotic-mediated process. Physiol. Plant..

[35] Sun S.J., Meng P., Zhang J.S., Wan X. (2011). Variation in soil water uptake and its effect on plant water status in *Juglans regia* L. during dry and wet seasons. Tree Physiol..

[36] Choat B., Jansen S., Brodribb T.J., Cochard H., Delzon S., Bhaskar R., Bucci S.J., Feild T.S., Gleason S.M., Hacke U.G. (2012). Global convergence in the vulnerability of forests to drought. Nature.

[37] Abdelraheem A., Esmaeili N., O’Connell M., Zhang J. (2019). Progress and perspective on drought and salt stress tolerance in cotton. Ind. Crop. Prod..

[38] Zafar M.M., Rehman A., Razzaq A., Parvaiz A., Mustafa G., Sharif F., Mo H., Youlu Y., Shakeel A., Ren M. (2022). Genome-wide characterization and expression analysis of Erf gene family in cotton. BMC Plant Biol..

[39] Petit G., Anfodillo T., Carraro V., Grani F., Carrer M. (2011). Hydraulic constraints limit height growth in trees at high altitude. New Phytol..

[40] Yang X.D., Anwar E., Xu Y.L., Zhou J., Sha L.B., Gong X.W., Ali A., Gao Y.C., Liu Y., Ge P. (2022). Hydraulic constraints determine the distribution of heteromorphic leaves along plant vertical height. Front. Plant Sci..

[41] Domec J.-C., Smith D.D., McCulloh K.A. (2017). A synthesis of the effects of atmospheric carbon dioxide enrichment on plant hydraulics: Implications for whole-plant water use efficiency and resistance to drought. Plant Cell Environ..

[42] Qaderi M.M., Martel A.B., Dixon S.L. (2019). Environmental Factors Influence Plant Vascular System and Water Regulation. Plants.

[43] Lauriks F., Salomon R.L., De Roo L., Steppe K. (2021). Leaf and tree responses of young European aspen trees to elevated atmospheric CO_2_ concentration vary over the season. Tree Physiol..

[44] Martinez-Vilalta J., Prat E., Oliveras I., Pinol J. (2002). Xylem hydraulic properties of roots and stems of nine Mediterranean woody species. Oecologia.

[45] Boughalleb F., Denden M., Tiba B.B. (2009). Anatomical changes induced by increasing NaCl salinity in three fodder shrubs, Nitraria retusa, Atriplex halimus and Medicago arborea. Acta Physiol. Plant.

[46] Creek D., Lamarque L.J., Torres-Ruiz J.M., Parise C., Burlett R., Tissue D.T., Delzon S. (2020). Xylem embolism in leaves does not occur with open stomata: Evidence from direct observations using the optical visualization technique. J. Exp. Bot..

[47] Chen Y.J., Choat B., Sterck F., Maenpuen P., Katabuchi M., Zhang S.B., Tomlinson K.W., Oliveira R.S., Zhang Y.J., Shen J.X. (2021). Hydraulic prediction of drought-induced plant dieback and top-kill depends on leaf habit and growth form. Ecol. Lett..

[48] Luo D., Wang C., Jin Y., Li Z., Wang Z. (2022). Different hydraulic strategies under drought stress between *Fraxinus mandshurica* and Larix gmelinii seedlings. J. For. Res..

[49] Shao J., Zhou X., Zhang P., Zhai D., Yuan T., Li Z., He Y., McDowell N.G. (2023). Embolism resistance explains mortality and recovery of five subtropical evergreen broadleaf trees to persistent drought. Ecology.

[50] Smith-Martin C.M., Muscarella R., Ankori-Karlinsky R., Delzon S., Farrar S.L., Salva-Sauri M., Thompson J., Zimmerman J.K., Uriarte M. (2022). Hurricanes increase tropical forest vulnerability to drought. New Phytol..

[51] Wang D.R., Venturas M.D., Mackay D.S., Hunsaker D.J., Thorp K.R., Gore M.A., Pauli D. (2020). Use of hydraulic traits for modeling genotype-specific acclimation in cotton under drought. New Phytol..

[52] Gattmann M., McAdam S.A.M., Birami B., Link R., Nadal-Sala D., Schuldt B., Yakir D., Ruehr N.K. (2023). Anatomical adjustments of the tree hydraulic pathway decrease canopy conductance under long-term elevated CO_2_. Plant Physiol..

[53] Lauriks F., Salomón R.L., De Roo L., Goossens W., Leroux O., Steppe K. (2022). Limited plasticity of anatomical and hydraulic traits in aspen trees under elevated CO_2_ and seasonal drought. Plant Physiol..

[54] Domec J.C., Schafer K., Oren R., Kim H.S., McCarthy H.R. (2010). Variable conductivity and embolism in roots and branches of four contrasting tree species and their impacts on whole-plant hydraulic performance under future atmospheric CO_2_ concentration. Tree Physiol..

[55] Hao G.Y., Holbrook N.M., Zwieniecki M.A., Gutschick V.P., BassiriRad H. (2018). Coordinated responses of plant hydraulic architecture with the reduction of stomatal conductance under elevated CO_2_ concentration. Tree Physiol..

[56] Tognetti R., Longobucco A., Raschi A. (1999). Seasonal embolism and xylem vulnerability in deciduous and evergreen Mediterranean trees influenced by proximity to a carbon dioxide spring. Tree Physiol..

[57] Warren J.M., Norby R.J., Wullschleger S.D. (2011). Elevated CO_2_ enhances leaf senescence during extreme drought in a temperate forest. Tree Physiol..

[58] Cai J., Tyree M.T. (2010). The impact of vessel size on vulnerability curves: Data and models for within-species variability in saplings of aspen, Populus tremuloides Michx. Plant Cell Environ..

[59] Cochard H., Froux F., Mayr S., Coutand C. (2004). Xylem wall collapse in water-stressed pine needles. Plant Physiol..

[60] Jiang X., Choat B., Zhang Y.-J., Guan X.-Y., Shi W., Cao K.-F. (2021). Variation in Xylem Hydraulic Structure and Function of Two Mangrove Species across a Latitudinal Gradient in Eastern Australia. Water.

[61] Zimmermann M.H. (1983). Xylem Structure and the Ascent of Sap.

[62] Martinez-Vilalta J., Cochard H., Mencuccini M., Sterck F., Herrero A., Korhonen J.F., Llorens P., Nikinmaa E., Nole A., Poyatos R. (2009). Hydraulic adjustment of Scots pine across Europe. New Phytol..

[63] Awad H., Barigah T., Badel E., Cochard H., Herbette S. (2010). Poplar vulnerability to xylem cavitation acclimates to drier soil conditions. Physiol. Plant..

[64] Plavcová L., Hacke U.G. (2012). Phenotypic and developmental plasticity of xylem in hybrid poplar saplings subjected to experimental drought, nitrogen fertilization, and shading. J. Exp. Bot..

[65] Beniwal R.S., Langenfeld-Heyser R., Polle A. (2010). Ectomycorrhiza and hydrogel protect hybrid poplar from water deficit and unravel plastic responses of xylem anatomy. Environ. Exp. Bot..

[66] Cao X., Jia J., Zhang C., Li H., Liu T., Jiang X., Polle A., Peng C., Luo Z.B. (2014). Anatomical, physiological and transcriptional responses of two contrasting poplar genotypes to drought and re-watering. Physiol. Plant..

[67] Robert E.M., Koedam N., Beeckman H., Schmitz N. (2009). A safe hydraulic architecture as wood anatomical explanation for the difference in distribution of the mangroves Avicennia and Rhizophora. Funct. Ecol..

[68] Schmitz N., Verheyden A., Beeckman H., Kairo J.G., Koedam N. (2006). Influence of a salinity gradient on the vessel characters of the mangrove species Rhizophora mucronata. Ann. Bot..

[69] Kondoh S., Yahata H., Nakashizuka T., Kondoh M. (2006). Interspecific variation in vessel size, growth and drought tolerance of broad-leaved trees in semi-arid regions of Kenya. Tree Physiol..

[70] Rosner S., Nöbauer S., Voggeneder K. (2021). Ready for Screening: Fast Assessable Hydraulic and Anatomical Proxies for Vulnerability to Cavitation of Young Conifer Sapwood. Forests.

[71] Hacke U.G., Sperry J.S., Pockman W.T., Davis S.D., McCulloh K.A. (2001). Trends in wood density and structure are linked to prevention of xylem implosion by negative pressure. Oecologia.

[72] Janssen T.A.J., Holtta T., Fleischer K., Naudts K., Dolman H. (2020). Wood allocation trade-offs between fiber wall, fiber lumen, and axial parenchyma drive drought resistance in neotropical trees. Plant Cell Environ..

[73] Chen Z., Zhu S., Zhang Y., Luan J., Li S., Sun P., Wan X., Liu S. (2020). Tradeoff between storage capacity and embolism resistance in the xylem of temperate broadleaf tree species. Tree Physiol..

[74] Li X., Kang S., Zhang X., Li F., Lu H. (2018). Deficit irrigation provokes more pronounced responses of maize photosynthesis and water productivity to elevated CO_2_. Agric. Water Manag..

[75] Pammenter N.v., Van der Willigen C. (1998). A mathematical and statistical analysis of the curves illustrating vulnerability of xylem to cavitation. Tree Physiol..

[76] Sperry J.S., Saliendra N.Z. (1994). Intra- and inter-plant variation in xyiem cavitation in Betula occidentalis. Plant Cell Environ..

[77] Choat B., Ball M.C., Luly J.G., Holtum J.A.M. (2005). Hydraulic architecture of deciduous and evergreen dry rainforest tree species from north-eastern Australia. Trees.

[78] Sperry J.S., Sullivan J.E.M. (1992). Xylem embolism in response to freeze-thaw cycles and water stress in ring-porous, diffuse-porous, and conifer species. Plant Physiol..

[79] R Core Team (2022). R: A Language and Environment for Statistical Computing.

[80] Duursma R.A., Choat B. (2017). fitplc: An R package to fit hydraulic vulnerability curves. J. Plant Hydraul..

